# Counselees’ Expressed Level of Understanding of the Risk Estimate and Surveillance Recommendation are Not Associated with Breast Cancer Surveillance Adherence

**DOI:** 10.1007/s10897-015-9869-x

**Published:** 2016-04-01

**Authors:** Akke Albada, Sandra van Dulmen, Henrietta Dijkstra, Ivette Wieffer, Arjen Witkamp, Margreet G. E. M. Ausems

**Affiliations:** 1 Department of Medical Genetics, University Medical Centre Utrecht, Utrecht, The Netherlands; 2 NIVEL (Netherlands Institute for Health Services Research, Utrecht, The Netherlands; 3 Department of Primary and Community Care, Radboud University Medical Centre, Nijmegen, The Netherlands; 4 Department of Health Sciences, Buskerud and Vestfold University College, Drammen, Norway; 5 Department of Surgery, University Medical Centre Utrecht, Utrecht, The Netherlands

**Keywords:** Breast cancer, Genetic counseling, Surveillance, Mammography, Adherence, Risk perception

## Abstract

We studied counselees’ expressed understanding of the risk estimate and surveillance recommendation in the final consultation for breast cancer genetic counseling in relation with their risk perception, worry and cancer surveillance adherence 1 year post-counseling. Consecutive counselees were included from 2008 to 2010. Counselees with an indication for diagnostic DNA-testing for themselves or a breast cancer affected relative were requested to complete online questionnaires before and after counseling and one year after counseling (*N* = 152–124). Self-reported surveillance was compared to surveillance recommendations. Consultations were videotaped. Counselees’ reactions to the risks and recommendations were coded. Statements about the risk perception and surveillance intentions of breast cancer unaffected counselees were transcribed. Associations with outcomes were explored. Almost all breast cancer unaffected counselees (>90 %) reacted to their risk estimate with an utterance indicating understanding and this reaction was not significantly associated with their post-visit risk perception alignment. Over one-third (38.6 %) overestimated their risk post-counseling. Few counselees (5.8 %) expressed surveillance intentions. One year after counseling, about three-quarters (74.0 %) of the breast cancer unaffected counselees had adhered to the surveillance recommendation. Almost one-quarter (23.3 %) had performed more mammograms/MRI scans than recommended, which was associated with prior mammography uptake (*n* = 47; *X*^*2*^ = 5.2; *p* = .02). Counselees’ post-counseling overestimation of their risk, high levels of worry and high surveillance uptake were not reflected in their reactions to the counselor’s information during the final visit.

**Electronic supplementary material:**

The online version of this article (doi:10.1007/s10897-015-9869-x) contains supplementary material, which is available to authorized users.

## Introduction

Breast cancer genetic counseling aims to promote informed decision making concerning surveillance of those at increased risk of (hereditary) breast cancer (Resta et al. 2008). Counselees can be referred by their general practitioner (GP) or consultant. Genetic counseling might entail one or more visits and a DNA-test. The genetic counselor provides a risk estimate and surveillance recommendation to help the counselee understand her risk and adapt to it. However, genetic counseling appears to have little impact on counselees’ perception of their own and their relatives’ risk (Butow et al. 2003; Meiser and Halliday 2002; Smerecnik et al. 2009); 22 to 50 % of counselees continue to overestimate their risk (Butow et al. 2003). Moreover, their worry about breast cancer remains substantial and might be related to a high frequency of breast self-examination (Van Dooren et al. 2003). Overestimation of their risk and high cancer worry might lead to higher use of breast surveillance than recommended. However, little is known about the adherence to the surveillance recommendation of a large group of counselees, namely those who were the first in their family to request breast cancer genetic counselling and in whom no BRCA1/2 gene mutation was detected (i.e., they received a BRCA1/2 negative test result) (Albada et al. 2014; Van Dijk et al. 2006). For these counselees and for those for whom no DNA-testing is performed, surveillance recommendation depends on the degree of their pedigree-based risk. A Dutch study found that almost all counselees with a BRCA1/2 negative test result intended to have a mammogram in the next year, but their uptake was not assessed (Van Dijk et al. 2005). An Australian study found that 86 % of counselees with a pedigree-based risk estimate had an uptake at the recommended or higher frequency (Meiser et al. 2001). Notably, overuse of surveillance has scarcely been assessed. Risk perception, past surveillance uptake and intention for surveillance have been identified as predictors of surveillance uptake (Lechner et al. 1997; Michie et al. 2002). Therefore, counselees’ overestimation of breast cancer risk (Smerecnik et al. 2009), their surveillance habits (Meiser et al. 2001) and high intention for surveillance (Van Dijk et al. 2005) might lead to more frequent mammograms than recommended. This is of concern because of the risks of early and repeated radiation exposure and the lack of sensitivity of mammography in young women (Heyes et al. 2009).

The counselees’ personal life story seems to influence their interpretation of the communicated risk estimate and might also predict overestimation of risks and surveillance uptake (Vos et al. 2012). Therefore, some researchers have recommended discussing the counselee’s risk perception in genetic counseling (Van Dijk et al. 2004a; Michie et al. 2003; Pieterse et al. 2006). However, studies have shown that counselees’ pre-existing risk perception and interpretation of the risk estimate were rarely discussed (Pieterse et al. 2006). Counselees did not react to most of the risk estimates given and this might be related to their low recall (Michie et al. 2005). To date, counselees’ reactions to the risk estimate in the final visit have not been a topic of investigation in studies. Counselees’ level of expressed understanding of this estimate and the surveillance recommendation might be associated with the alignment of their post counseling risk perception and adherence. Moreover, counselees might express their surveillance intention. Counselors can only tailor their information about pros and cons of surveillance if counselees express their (mis)understanding of the risk estimate and recommendation and if they share their surveillance intentions. This type of discussion might enhance the quality of an informed decision. Also, it might enhance counselees’ communication of the risk estimates to their relatives. Moreover, behavior change (i.e., starting or stopping periodic surveillance) is facilitated by discussing how the change may be undertaken (Gollwitzer and Sheeran 2006). Spelling out the *when*, *where* and *how* of the intended behavior is theorized to increase adherence. The environmental and contextual cues of these details are expected to prompt the desired behavior. Discussions of the counselee’s intentions might thus help the counselee to realize the recommended surveillance uptake.

### Purpose of the Study

In this paper we report on a study of the counselees’ expressed understanding as a response to the risk estimate and surveillance recommendation and whether they express surveillance intentions in the final consultation for breast cancer genetic counseling. Associations between the level of understanding, counselees’ prior surveillance behavior and intentions with risk perception alignment and surveillance adherence 1-year post counseling were explored.

## Methods

### Design

The present study comprises a secondary analysis of data obtained as part of a larger study on breast cancer genetic counseling at the department of Medical Genetics of the University Medical Centre Utrecht (UMCU) (Albada et al. 2012). The study was approved by the medical ethical committee of this hospital. The department of Medical Genetics offers breast cancer genetic counseling according to the Dutch guidelines (CBO 2008). Consecutive new counselees were included from February 2008 to April 2010. Female counselees aged 18 years or older and who were the first of their first degree family members to seek breast cancer genetic counseling, were sent information about the study and an opt-out form. A few counselees were ineligible because of lack of internet or email access (24 of 371; 6.5 %; supplementary material Fig. 1). At the start of the consultation the counselor collected the informed consent form. Both first and final consultations were videotaped with an unmanned camera directed at the counselor. After several weeks, a summary letter of the final consultation was sent to the counselee. Counselees completed a digital questionnaire before counseling, approximately 1 week after the final visit and approximately 1 year after the final visit.

All 14 counselors performing breast cancer genetic counseling consultations at the department participated and counseled 2 to 25 counselees each. Counselors were clinical geneticist (*n =* 3), resident in clinical genetics (*n =* 5), genetic counselor (*n =* 3) or genetic counselor in training (*n =* 3). Most were female (*n =* 2 male).

### Sample

The current paper reports on 162 female counselees who received an indication for diagnostic DNA-testing for themselves or a breast cancer affected relative and/or a follow-up consultation. For 9 counselees the follow-up consultation was not videotaped due to logistic failure. For one counselee the single first consultation was not videotaped due to logistic failure and there was no follow-up consultation. Thus, the results concerning the risk communication are based on 152 counselees. Data were initially gathered for a randomized controlled trial of the effects of a pre-visit tailored website on genetic counseling outcomes for hereditary breast cancer in which participants were randomized to receive usual care or usual care plus an educational website. In the current study, this group allocation was controlled for.

### Instrumentation and Procedures

#### Counselee Characteristics

Having children, family cancer history and educational attainments were assessed in the baseline counselee questionnaire. The family (cancer) history was derived from the medical file if missing. Two missing values on education were imputed with the median. The breast cancer disease status (affected/unaffected), referral pathway (by GP/medical consultant), indication for DNA-test and test uptake were derived from the medical file. Counselors rated the counselees’ risk to (re-)develop breast cancer in the future on a scale from 0 to 100 % in this questionnaire. Their estimation was based on the Claus tables and the Claus extended formula (Van Asperen et al. 2004). When the risk estimate was revised due to, for instance, changes in the family cancer history or newly available DNA-test results, we used the revised risk estimate from the medical file. For five breast cancer affected counselees with a BRCA1/2 negative DNA-test result this estimate was missing and was imputed with the mean estimate for affected counselees with indication for testing and a BRCA1/2 negative test result.

#### Counselee Questionnaires

Counselees rated their *perceived risk* to (re-)develop breast cancer in the future and this risk for their first degree female relatives (FDFR) on visual analogue scales from 0 to 100 %. Risk perception was defined as aligned if the counselee and counselor estimates were within the same risk category [e.g. population or slightly increased risk (<20 %), moderate (20–30 %) or high risk (≥30 %) of developing breast cancer (again)]. This measure was chosen because the recommendation for surveillance is based on these risk categories (Van Dijk et al. 2004a; CBO 2008).

The *breast cancer worry* in the past 2 weeks was assessed with the Cancer Worry Scale (Van Oostrom et al. 2007). Three questions assessed the frequency of worry about breast cancer, influence on mood, and interference with daily activities with 4-point response categories (almost never to almost always). One item assessed the extent of the worry (1not at all to 4 very much).

One question assessed how often a woman had performed *breast self-examination* (BSE) in the past year. The answer categories were: no, less than once a month, monthly, weekly, daily, and not applicable because of risk reducing breast surgery (Van Dijk et al. 2004b).

*Intention* and adherence to surveillance recommendations are only reported for breast cancer unaffected counselees. Affected counselees receive surveillance recommendations during regular follow-up visits with their surgeon based on national guidelines (CBO 2008). The post-visit questionnaire assessed the intention for adherence to the surveillance recommendation with the following question, “Do you think you will have a mammogram every year or at least once every 2 years if indicated?” (Van Dijk et al. 2005). The questionnaires 1 week and 1 year after the final consultation assessed whether the counselee had risk reducing breast surgery and whether a woman had a mammogram and/or MRI scan in the last year (no/yes; if yes, how many times and in which month and year) (Van Dijk et al. 2004b). *Adherence to surveillance recommendations* was determined by comparing the uptake of mammography and/or MRI in the last year to the age and risk specific surveillance recommendations. These recommendations were based on the Dutch breast cancer guideline (CBO 2008; Supplementary Appendix A). The medical file was checked to see if the counselor’s recommendation differed from the guideline recommendation. For some counselees the counselor had recommended surveillance from a younger age, and this was incorporated in the determination of these counselees’ adherence. If the recommendation was to have a mammogram once every 2 years, the mammography uptake of the year previously, reported in the questionnaire after the final consultation, was also considered. Adherence to surveillance recommendations was scored as *one* in case of agreement, and *zero* in case of no agreement. Non-adherence was specified as underuse of surveillance if the counselee had performed no surveillance despite the counselor’s positive advice given the counselee’s age and risk. If the counselee had performed more mammograms/MRI than recommended or started at an earlier age than recommended, this was coded as overuse of surveillance.

#### Coding of the Videotaped Consultations

The consultations were rated with the RIASgene (Albada et al. 2012), an adaptation for the genetic counseling setting of Roter’s Interaction Analysis System (RIAS) (Roter 2006). This RIAS coding has been shown to have high interrater reliability (ICC = .86) (Albada et al. 2014). The RIASgene coding was used to identify counselors’ utterances of the risk estimate and breast surveillance recommendation for both the counselee and her relatives. The third author (HD) transcribed the counselee’s reaction to these utterances and rated: (1) misunderstanding, (2) general understanding or acceptance, (3) clear understanding (i.e. a rehearsal of the risk or recommendation in the counselee’s own words or a reflection on its meaning). Both the wording and tone of voice were taken into account when coding the reaction. We determined whether the counselee had given at least one utterance indicating clear understanding, general understanding and an indication for misunderstanding (1/0). Additionally, HD checked whether the counselee had said something about her view on her risk perception, transcribed it, and coded whether it was concordant with the risk estimate. Furthermore, intentions about surveillance were transcribed and scored for whether they included details concerning where, when and how surveillance would be completed.

### Data Analyses

A variable was created for whether a counselee had uttered at least one clear understanding of the risk estimate or a statement of concordant risk perception (yes/no). Associations with risk perception alignment, breast cancer worry and adherence to breast surveillance recommendations were explored with Chi-square tests. Associations between prior mammography uptake and adherence and prior breast self-examination (BSE) uptake and post counseling BSE uptake were explored with Chi-square tests. Additionally, adherence to breast surveillance recommendation was the dependent variable in a multilevel regression equation for unaffected counselees with a positive surveillance recommendation. This regression was also performed for unaffected counselees with a recommendation not to perform surveillance. In both regression equations the following dependent variables were determined a priori: past uptake, risk perception alignment, intention, whether a surveillance recommendation was discussed and intervention allocation. Intervention allocation had no significant association (Chi^2^ test) with any of the outcome variables. Counselees were nested within counselors. Analyses were performed with Stata 11.

## Results

### Sample Characteristics

The response rate was 58.6 %. Half of the decliners gave a reason (72/139; 50.4 %, see flowchart Fig. 1). Most preferred the visit not be videotaped (48/72; 66.7 %). There were no significant differences between participants and decliners in age, disease status, family history of cancer and referral pathway. Most (*n* = 97) counselees had a follow-up consultation, and 65 counselees had a single consultation (Table 1). For 45.7 % of the counselees there was an indication for DNA-testing of themselves, and these were primarily breast cancer affected counselees. For 48.8 % of the counselees there was an indication for DNA-testing for a relative. For 53 (70.7 %) of the latter counselees, testing was not performed because the affected relative was not (yet) willing or able to be tested, was not requested to be tested by the counselee or was deceased. For the counselees for whom a DNA-test was performed, 81.2 % received a BRCA1/2 negative test result. Counselees for whom no DNA-test was performed, and those receiving a BRCA1/2 negative test result, received a pedigree based risk estimate. None of the counselees had undergone prophylactic breast surgery pre-counseling.

**Table 1:** Counselee characteristics

Age (years)***	Unaffected (*n* = 89)		Affected (*n* = 73)		Total (*N* = 162)	
	M	Sd	M	Sd	M	Sd
	37.9	10.6	47.5	10.3	42.2	11.5
	*n*	%	*n*	%	*n*	%
Children (having children)**	52	58.4	60	82.2	112	69.1
Education^a^:						
<High school level	2	2.3	0	0.0	2	1.3
High school/ Secondary education	26	29.9	19	26.8	45	28.5
Middle vocational education	25	28.7	23	32.4	48	30.4
University (MSc/BSc)/higher vocational education (BSc)	34	39.1	29	40.9	63	39.9
Referred by GP (vs. consultant)***	65	73.0	11	15.1	76	46.9
Follow-up consultation (vs. only one consultation)***	32	36.0	65	89.0	97	59.9
Indication for DNA-testing^b^	83	93.3	70	95.9	153	94.4
For counselee	8	9.0	66	90.4	74	45.7
Test uptake	3	–	59	–	62	–
For relative	75	84.3	4	5.5	79	48.8
Test uptake	22	–	1	–	23	
BRCA1/2-test result^c^						
BRCA1/2 negative	21		48		69	42.6
BRCA1/2 mutation carriers	NA	NA	7		7	4.3
50 % risk of being a BRCA1/2 mutation carrier^d^	3		NA	NA	3	1.9
VUCS ^e^	1	1.1	6	8.2	7	4.3
Breast cancer risk category counselee**						
Population (<20 % lifetime risk)	35	39.3	44	60.3	79	48.8
Moderate (20–30 % lifetime risk)	42	47.2	15	20.6	57	35.2
High (≥30 % lifetime risk)	12	13.5	14	19.2	26	16.1
Breast cancer risk category FFDR						
Population (<20 % lifetime risk)	37	41.6	35	48.0	72	44.4
Moderate (20–30 % lifetime risk)	38	42.7	25	34.3	63	38.9
High (≥30 % lifetime risk)	14	15.7	13	17.8	27	16.7

^a^4 missing values; ^b^for 10 counselees there was a second consultation to determine whether there was an indication for DNA-testing and medical file data from relatives showed that there was none, for 4 of these counselees the counselor had indicated that there was an indication for DNA-testing after the first consultation; ^c^One test result indicated a BRCA1/2-mutation as well as an unclassified variant; ^d^breast cancer unaffected counselees with a first degree relative who tested positive for a BRCA1/2 mutation; ^e^Variant of Unknown Clinical Significance; Significant difference between breast cancer affected and unaffected counselees; t-test and X^2^ ** *p* < .01 ***p < .001

We conducted a validity check on the self-reported surveillance uptake by comparing this to medical records. Ten breast cancer unaffected counselees had surveillance in the UMCU and had completed the question regarding mammography/MRI uptake. For all of them their self-reported surveillance uptake was confirmed by the surveillance uptake as registered (data not shown). Seven of these counselees had performed surveillance, and this was initiated because of increased risk.

### Inter-Rater Agreement

All possible expressions of an intention were discussed by HD and the first author (AA). For 74 final visits the level of agreement was coded by two coders independently. The percentage of agreement between the raters for whether the counselee had expressed clear understanding varied from 70 to 85 %, for whether the counselee had expressed understanding agreement ranged from 75 to 91 %, and for misunderstanding it ranged from 83 to 96 %. When the coders did not agree, they discussed the response and again viewed the video, if needed, to reach consensus.

### Risk Communication

Twenty-three counselees had not received a personal breast cancer risk estimate in their final consultation (17 unaffected and 6 affected, Table 2). This was often because further medical information about relatives was needed or there was an indication for DNA-testing of a relative but this test was not performed. Approximately one-quarter (24.6 %) of the breast cancer unaffected counselees, and 35.9 % of the affected counselees, uttered clear *understanding of the risk estimate*. Twenty-five counselees expressed something about their view on their risk perception in the consultation. For four of them, their expressed view signaled a misunderstanding or unexpectedness of the risk estimate given by the counselor; for the others their view signaled understanding of the estimate (not in Table). Four of the five counselees who had uttered a misunderstanding of their risk estimate or a discordant risk perception had misaligned risk perceptions 1 year post-counseling.

**Table 2:** Risk estimation and surveillance recommendations and the percentages of visits in which the counselee responded with at least one utterance indicating understanding or misunderstanding (*N* = 152)^a^

	Unaffected counselees (*n*=82)				Affected counselees (*n*=70)			
	Visits with risk estimate	Ce response in visits including a risk estimate*			Visits with risk estimate	Ce response in visits including a risk estimate*		
		Clear understanding	Understanding	Misunderstanding		Clear understadning	Understanding	Misunderstanding
	*n* (%)	*n* (%)	*n* (%)	*n* (%)	*n* (%)	*n* (%)	*n* (%)	*n* (%)
Communica-tion of risk estimate counselee	65 (79.3)**	16 (24.6)	60 (92.3)	1 (1.5)	64 (91.4)	23 (35.9)	60 (93.8)	1 (1.6)
Communication of risk estimate FFDR	45 (54.9)	7 (15.6)	41 (89.1)	1 (2.2)	44 (62.9)	12 (27.3)	41 (93.2)	0 (0.0)
	Advice discussed	Clear understanding	Understanding	Misunderstanding		Clear understadning	Understanding	Misunderstanding
		% of visits with advice				% of visits with advice		
Advice about breast surveillance counselee	50 (61.0)	20 (40.0)	45 (90.0)	5 (10.0)	–	–	–	–
Advice about breast surveillance FFDR	19 (23.2)**	4 (21.1)	15 (79.0)	1 (5.3)	42 (60.0)	15 (35.7)	36 (85.7)	2 (4.8)

*Cr* counselor, *ce* counselee

^a^ 10 Missing cases due to logistic failure to record the final consultation

*Categories are not mutually exclusive, counselees have given more than one type of response

**Significant difference between breast cancer affected and unaffected counselees *p* < .05

- Not Applicable as no advice for surveillance is given to breast cancer affected counselees

Forty percent of the unaffected counselees uttered a clear *understanding of the surveillance recommendation*. Of the four unaffected counselees who uttered a disagreement with the surveillance recommendation, two were adherent 1 year post-counseling. Nine (5.8 %) counselees expressed a surveillance intention (supplementary material: Fig. 2). Six of them were breast cancer unaffected. Four counselees expressed an intention to perform mammogram/MRI to adhere to the recommendation, and three of them were adherent. One counselee expressed an intention for mammography uptake at an earlier age than recommended, and she had indeed performed a mammogram 1 year post-counseling. Four intentions included information about when to perform the surveillance, and two included information about where or with which health care provider to arrange the surveillance.

### Risk Perception and Adherence

Immediately post-counseling about half of the unaffected counselees (48.6 %) accurately perceived their *breast cancer risk* and 38.6 % overestimated their risk (Table 3). More than half of the affected counselees (65.5 %) correctly estimated the risk for their FDFR. At 1 year post counseling there were significantly less counselees with an accurate risk perception than 1 week post-counseling (*X*^*2*^ = 13.70; *p* = .000). Most unaffected counselees (55.1 %) overestimated their risk. More than one-third of the affected counselees (35.0 %) overestimated the risk for their FDFR. Having received a personal risk estimate in the final visit was not significantly related to the risk perception alignment for themselves (*X*^*2*^ = .33; *p* = .57). Also, there was no significant association between the utterance of a clear understanding or concordant risk perception in the visit and the alignment of risk perception post-visit (*X*^*2*^ = 1.17; *p* = .28).

**Table 3:** Risk perception alignment post counseling and 1 year after counseling

	Total	Accurate perception		Overestimation		Underestimation	
	*n*	*n*	%	*n*	%	*n*	%
After genetic counseling(*n* = 124)							
Breast cancer unaffected counselees^a^	70	34	48.6	27	38.6	9	12.9
Br risk <20^c^	28	15	53.6	13	46.4	–	–
Br risk 20–30	32	12	37.5	14	43.8	6	18.8
Br risk >30^b,c^	8	5	–	–	–	3	–
50 % risk of being BRCA1/2 mutation carrier^b^	2	2	–	0	–	0	–
Breast cancer affected counselees*	55	36	65.5	9	16.4	10	18.2
Br risk <20^c^	25	21	84.0	4	16.0	–	–
Br risk 20–30	20	10	50.0	5	25.0	5	25.0
Br risk >30^b,c^	4	2	–	–	–	2	–
BRCA1/2 mutation carriers^b^	6	3	–	0	–	3	–
One year after the final visit (*N* = 142)							
Breast cancer unaffected counselees ^a^	78	27	34.6	43	55.1	8	10.3
Br risk <20^c^	30	8	26.7	22	73.3	–	–
Br risk 20–30	38	14	36.8	21	55.3	3	7.9
Br risk >30^b,c^	8	4	–	–	–	4	–
50 % risk of being BRCA1/2 mutation carrier^b^	2	1	–	0	–	1	–
Breast cancer affected counselees*	60	31	51.7	21	35.0	8	13.3
Br risk <20^c^	29	19	65.5	10	34.5	–	–
Br risk 20–30	22	5	22.7	11	50.0	6	27.3
Br risk >30^b,c^	3	3	–	–	–	0	–
BRCA1/2 mutation carriers^b^	6	4	–	0	–	2	–

^a^Including counselees who received an indication for DNA-testing for a relative but no testing was performed, counselees received an uninformative result and counselees who received a VUCS

^b^Because of low numbers no percentages are displayed

^c^Underestimation of risk category is not possible for counselees in the lowest risk category and overestimation of risk category not possible for counselees in highest risk category

* For breast cancer affected counselees their perception of the risk for their first degree female relatives was compared to the counselor’s estimate of this risk

Counselees’ *breast cancer worry* had lowered from pre- to post-counseling (1.75 vs. 1.64; *t* = 2.51; *p* = 0.01) and from pre- to 1 year post-counseling (1.75 vs. 1.64; *t* = 2.79; *p* = .006). Their breast cancer worry was not significantly associated with expressed understanding of the risk estimate at any of the time points.

Less than one-tenth of the breast cancer unaffected counselees performed more than monthly *BSE* (Table 4). Of the breast cancer affected counselees, more than one-fifth performed more than monthly BSE. Post counseling, counselees intended to perform BSE more frequently than they had done pre-counseling (*n* = 99; *X*^*2*^ = 43.6; *p* = .000) and 1 year post-counseling the uptake was higher than pre-counseling (*n* = 128; *X*^*2*^ = 27.4; *p* = .000).

**Table 4:** Breast self-examination of breast cancer unaffected and affected counselees before genetic counseling and 1 year after their final visit (*n* = 158^a^)

		Weekly or more frequently		Monthly		< Monthly		Never	
	*n*	*n*	%	*n*	%	*n*	%	*n*	%
Before genetic counseling									
Breast cancer unaffected counselees	87	6	6.9	33	37.9	36	41.4	12	13.8
Br risk <20	33	2	6.1	15	45.5	13	39.4	3	9.1
Br risk 20–30	41	3	7.3	13	31.7	19	46.3	6	14.6
Br risk >30	10	1	10.0	4	40.0	4	40.0	1	10.0
50 % risk of being BRCA1/2 mutation carrier^b^	3	0	–	1	–	0	–	2	–
Breast cancer affected counselees	71	11	15.5	20	28.2	27	38.0	13	18.3
Counselees with a pedigree based risk estimation^c^	64	11	17.2	17	26.6	25	39.1	11	17.2
BRCA1/2 mutation carriers^b^	7	0	–	3	–	2	–	2	–
One year after the final visit									
Breast cancer unaffected counselees	74	7	9.5	34	46.0	23	31.1	10	13.5
Br risk <20	28	2	7.1	12	42.9	10	35.7	4	14.3
Br risk 20–30	36	3	8.3	19	52.8	10	27.8	4	11.1
Br risk >30^b^	8	1	–	3	–	3	–	1	–
50 % risk of being BRCA1/2 mutation carrier^b^	2	1	–	0	–	0	–	1	–
Breast cancer affected counselees	57	13	22.8	23	40.4	16	28.1	5	8.8
Counselees with a pedigree based risk estimation^c,d^	53	13	24.5	21	39.6	15	28.3	4	7.6
BRCA1/2 mutation carriers^b,d^	4	0	–	2	–	1	–	1	–

^a^Four missing values because the counselee did not complete the question about breast self-examination

^b^Because of low numbers no percentages are displayed

^c^Mostly after an uninformative DNA-test result

^d^For two breast cancer affected counselees with a pedigree based risk estimation and two carriers, breast self-examination was non-applicable in the questionnaire 1 year post counseling because of a performed bilateral prophylactic mastectomy, these counselees are not included in the results at 1 year after the final visit

Almost half of the breast cancer unaffected counselees had a *mammogram* in the year before their first consultation (*n* = 42; 48.3 %). Post counseling almost all unaffected counselees (*n* = 73; 91.3 %) intended to have a mammogram/MRI if this was indicated. One year after the final consultation, almost three quarters of breast cancer unaffected counselees (74 %) adhered to the genetic counselor’s recommendation (Table 5). Of the non-adherent counselees, 26 had a mammogram/MRI scan while this was not recommended by the counselor. Most of them (*n* = 19) were younger than the recommended age to start mammography given their risk category. Others (*n* = 7) had an uptake of two instead of one mammograms/MRI scans in 1 year. Two counselees had not performed a mammogram/MRI scan while this was recommended. There were no significant differences in adherence between the unaffected counselees who had received BRCA1/2 negative test result from their affected relative (*n* = 17) and those in whom no DNA-testing was performed in relatives (*n* = 49). No significant associations were found between expressed a clear understanding of the surveillance recommendation and adherence (*X*^*2*^ = .05; *p* = .83). In bivariate analysis, the overuse of surveillance of counselees with a negative surveillance recommendation was associated to prior mammography uptake (*n* = 47; *X*^*2*^ = 5.2; *p* = .02). In the multilevel multivariate regression analysis, no significant associations with adherence were found.

**Table 5:** Adherence to breast surveillance recommendations of breast cancer unaffected counselees in breast cancer genetic counseling 1 year after their last visit (*n* =75)^a^

		Adherence	
	*n*	*n*	%
Br risk <20 %	28	22	78.6
Age <50 ^b,c^	27	21	77.8
50–75^d^	1	1	–
Br risk 20–30 %	36	26	72.2
Age <40 ^b,c^	21	14	66.7
40–50	13	11	84.6
50–75^d^	2	1	–
Br risk >30 %	7	4	57.1
Age <35^b,c,d^	3	2	–
35–60^d^	3	1	–
60–75^d^	1	1	–
50 % risk of being BRCA1/2 mutation carrier^d,e^	2	2	–
Total	73	54	74.0

^a^Including counselees who received an indication for DNA-testing for a relative but no testing was performed, counselees received an uninformative result and counselees who received a VUCS. 14 missing values because the counselee did not complete the question about surveillance uptake

^b^Age categories differ per risk category based on the age and risk specific surveillance recommendations according to the Dutch Breast Cancer Guideline, see Supplementary Appendix A

^c^There were no counselees older than 75 in this risk group

^d^Because of low numbers no percentages are displayed

^e^The two counselees for whom a mutation was found in a relative were in the age of surveillance recommendation (25–75 years of age)

## Discussion

This study is the first to explore counselees’ expressed understanding of the risk estimate and surveillance recommendation in the final consultation for breast cancer genetic counseling counselees. Most counselees responded affirmatively to the disclosure of their risk estimate and the surveillance recommendation. This might have given counselors the impression that the counselee perceived her risk in accordance with the estimate given and would follow-up on the surveillance recommendation. However, in line with earlier findings (Butow et al. 2003; Smerecnik et al. 2009), a large percentage of counselees overestimated their risk post counseling. The current study showed that counselees’ expressed understanding of their risk estimate was not significantly associated with the post-counseling risk perception alignment and breast cancer worry. More elaborate exploration of counselees’ beliefs about their risk might be needed to detect this lack of alignment between counselees’ and counselors’ views during the final visit.

Our results suggest that surveillance uptake is high in the Netherlands, at least for counselees with BRCA1/2 negative test-result or with no available DNA-test results for their affected relative. There were only two counselees with lower surveillance uptake than recommended. Notably, one-quarter of the counselees performed more surveillance than recommended. These were mainly counselees at slightly or moderately increased risk who started periodic surveillance before the recommended age of 50 or 40 years, respectively. The intention for mammography/MRI and overuse of surveillance compared to the recommendation seem unassociated with whether counselees’ expressed (mis)understanding in the final visit. Furthermore, surveillance intentions were scarcely expressed and included little information about the when, where and how of the intended behavior. This might have lead counselors to believe that the counselee intended to follow up on the surveillance recommendation. Hence, more explicit exploration of misunderstandings might be needed to explore intentions.

The need for a more elaborate discussion about counselees’ risk perception in the final visit is underlined by a significant decrease in accurate risk perception in the year post counseling. This might indicate that counselees’ perception of their risk drifts further away from the risk estimate given by the genetic counselor. Studies with shorter follow-up periods did not find significant decreases in risk perception (Pieterse et al. 2011; Van Dijk et al. 2005; Van Dijk et al. 2006). For the majority of breast cancer unaffected counselees in the current study, the indicated DNA-test was not performed by their affected relative. Possibly, in the absence of a DNA-test result, counselees are not reassured by a pedigree-based low risk estimate and this lack of reassurance increases in the year post counseling. For counselees with a negative BRCA1/2 test result inaccurate beliefs about this test result might be related to poor adjustment (Van Dijk et al. 2006). Counselors might thus additionally invite these counselees’ views about the value of their test results in an attempt to better align the counselees’ risk perception with the risk estimate.

Studies of genetic counseling in the USA and Australia found lower surveillance uptake of counselees than recommended (Botkin et al. 2003; Lerman et al. 2000; Meiser et al. 2001) and this might reflect international differences in the accessibility of mammography. In the Netherlands, surveillance is covered by (obligatory) health insurance and the average travel distance to the nearest hospital is small. Surveillance is dealt with by either a familial cancer center (Van Dijk et al. 2005), the surgeon, GP or the National Surveillance Program. These health system characteristics might lead to relatively high surveillance uptake.

Overuse of surveillance after genetic counseling was not previously described for counselees receiving a pedigree-based risk assessment, with or without a BRCA1/2 negative test result. Lerman et al. (2000) found that 30 % of non-carriers after predictive BRCA testing had an annual mammogram despite this not being recommended. Michie et al. (2002) found a similar desire to carry on with surveillance among counselees for predictive FAP testing who received a low risk result. Counselees felt that test results might be uncertain and reported little confidence in a genetic test based on a blood sample (Michie et al. 2003). This may be especially true when the test was performed in an affected relative, as shown by Van Dijk et al. (2008). However, counselees performing regular mammograms earlier than recommended might not be aware of radiation risks and the suboptimal sensitivity of mammography in the dense breast tissue of young women (Heyes et al. 2009).

As a referral is needed for mammography in the Netherlands, these women were probably referred by their GP. A Dutch study showed that GPs referred women for a mammogram despite a negative surveillance recommendation from the geneticist (De Bock et al. 2001). The GPs’ deviation from the genetic advice seems understandable as women might appreciate a mammogram more than reassurance (De Bock et al. 2001), and patients have been reported to put pressure on their GP to receive a referral (Burke et al. 2009). Also, referring physicians have been found to underestimate the radiation risks and did not inform patients about these risks (Ricketts et al. 2013). As mammography is covered by the health insurance system in the Netherlands and in many other countries, overuse could be included in discussions about health care costs. Opportunities to promote informed decision making regarding mammography uptake thus occur in both genetic counseling and GP practice (Sollie et al. 2015). Counselors’ information about (dis)advantages of mammography and explicit invitation of opposing views might enable counselees to share their surveillance intention.

### Study Limitations and Research Recommendations

This study had several limitations. First, the questionnaire assessed counselees’ intention to perform a mammogram/MRI scan if it was recommended by the counselor. However, not all counselees received such a recommendation as for some, periodic surveillance was not indicated. Follow-up studies should assess intention in relation to the recommendation received. Second, we cannot be sure whether some cases of reported overuse of surveillance occurred because of breast symptoms (e.g., a lump). Third, our questionnaire did not include questions about how a referral for mammography/MRI was obtained. However, as a large majority of the breast cancer unaffected counselees was referred to genetic counseling by their GP, we assumed that these counselees also obtained their referral for a mammogram from the GP. Finally, as only nine counselees expressed their intention, we lacked power to statistically explore an association with adherence.

### Practice Implications

This study showed that counselees’ risk perceptions and surveillance intentions are infrequently discussed in the final visit for breast cancer genetic counseling in the Netherlands. More elaborate discussion of these issues could enable counselors to discuss the disadvantages of periodic mammography, such as radiation risks and lack of sensitivity in young women. This might enable counselees to make a more informed decision. Moreover, motivational interviewing techniques could help counselors to discuss counselees’ motivation and intentions (Rollnick et al. 2010). This would mainly involve exploration and inviting counselees to elaborate on their attitude towards and feelings about the surveillance. Furthermore, prior mammography uptake might be associated with overuse of surveillance. Therefore, if a GP is considering sending a young woman for a mammogram based on the cancer family cancer history, the woman should be referred to genetic counseling first. Early referral to genetic counseling might help to prevent establishing a routine of regular mammography uptake without medical rationale.

## Electronic supplementary material

Below is the link to the electronic supplementary material.

ESM 1 (DOC 96 kb)

ESM 2 (DOC 29 kb)

ESM 3 (DOC 27 kb)
