# Supplementary material for: Counselees’ Expressed Level of Understanding of the Risk Estimate and Surveillance Recommendation are Not Associated with Breast Cancer Surveillance Adherence
Source: J Genet Couns. 2016 Apr 1;25(2):279–89. doi: 10.1007/s10897-015-9869-x (PMC4799246; doi:10.1007/s10897-015-9869-x)
Supplement: Supplementary file 1 — (DOC 96 kb) [file 10897_2015_9869_MOESM1_ESM.doc]

428 consecutive new counselees for breast cancer genetic counseling were sent information about the study with their appointment letter

371 counselees had their appointment in the inclusion period

Participating counselees in research on the initial consultations (n=197)

Counselees with an indication and/or a follow-up consultation (n=162)

T3: Complete data concerning expression of agreement with risk estimate and surveillance advice and expression of intentions (n=152), risk perception alignment T3 (n=124), intention for mammography/MRI uptake for unaffected counselees (n=80) and prior mammography uptake for unaffected counselee (n=87)

57 counselees cancelled or postponed their appointment or were counselled at a community hospital

35 counselees did not fulfil the inclusion criteria

139 counselees returned opt out forms, told the researcher by phone they did not wish to participate or told the counselor at the start of the initial consultation

24 did not have internet access

7 had a first degree relative who had attended breast cancer genetic counseling

2 were under 18 years

2 were referred because of ovarian cancer risk

7 did not want to fill in questionnaires

48 did not want to be videotaped

10 considered the study too much of a burden because of their cancer treatment or psychosocial situation

5 indicated lack of time as a reason

67 did not wish to give a reason

2 indicated another reason

336 counselees fulfilled the inclusion criteria

For 35 counselees there was no indication for DNA-testing in the family and no follow-up consultation

T4: Complete data concerning adherence based on surveillance uptake for unaffected counselees (n=75), breast self examination (n=158)

14 unaffected counselees failed to fill in the question on mammography/MRI uptake and 4 counselees failed to fill in questions about breast self-examination

10 final consultations were not videotaped due to logistic failure (9 follow-up consultations and one single consultation)

38 counselees failed to fill in questions about risk perception, 9 unaffected counselees failed to fill in the question about intention for mammography/MRI uptake and two counselees did not complete the question about prior mammography uptake

**Figure 1.** Flow chart
