# Supplementary material for: Counselees’ Expressed Level of Understanding of the Risk Estimate and Surveillance Recommendation are Not Associated with Breast Cancer Surveillance Adherence
Source: J Genet Couns. 2016 Apr 1;25(2):279–89. doi: 10.1007/s10897-015-9869-x (PMC4799246; doi:10.1007/s10897-015-9869-x)
Supplement: Supplementary file 2 — (DOC 29 kb) [file 10897_2015_9869_MOESM2_ESM.doc]

**Figure 2**  Examples of quotes of counselees’ reactions

**Reactions to the risk estimate**

**Clear understanding:**

*Counselees at population or slightly increased risk of breast cancer:*

“So everything’s normal” [101]

 “Yes, but not much increased (risk).” [266]

*Counselees at higher than 20% lifetime risk of breast cancer:*

“Normally it’s about ten percent, so let’s say it’s increased.” [46]

“One out of nine is already a high risk in my perception and this risk would be even higher.” [220]

*Counselees at higher than 30% risk*

“So, it (the outcome) is wrong.” [233]

**Understanding:**

*Counselees at population or slightly increased risk of breast cancer:*

“That’s a relief.” [336]

*Counselees at higher than 20% lifetime risk of breast cancer:*

 “Okay, yes, that’s a disappointment” [112]

**Misunderstanding:**

“And that was about ten to 15 percent you said.” [218]

**Reactions to recommendations for surveillance**

**Clear understanding**

*Counselees at population or slightly increased risk of breast cancer:*

“Yes, that’s what (the surveillance) I’m doing anyway.” [101]

*Counselees at higher than 20% lifetime risk of breast cancer:*

“Well that’s better than (starting surveillance) at 50 years of age.” [207]

**Understanding:**

*Counselees at population or slightly increased risk of breast cancer:*

 “Yes, well, that’s all right.” [267]

**Misunderstanding:**

*Counselees at population or slightly increased risk of breast cancer:*

“Okay, but I am under surveillance since several years, so we can’t do that anymore.” [218]

*Counselees at risk 20-30%*

“Not earlier (than that age)?” [308]

*Counselees at higher than 30% lifetime risk of breast cancer:*

“Surveillance? No risk reducing surgery?” [269]

**Expression of intentions**

 Examples of intentions for **surveillance according to the recommendation** (lifetime risk >30%)**:**

“I’ll just do that.” [269]

 Example of an intention for **surveillance from a younger age than recommended** (lifetime risk 20-30%):

“For now I would indeed opt for surveillance as of 35.” [192]
