# Supplementary material for: Counselees’ Expressed Level of Understanding of the Risk Estimate and Surveillance Recommendation are Not Associated with Breast Cancer Surveillance Adherence
Source: J Genet Couns. 2016 Apr 1;25(2):279–89. doi: 10.1007/s10897-015-9869-x (PMC4799246; doi:10.1007/s10897-015-9869-x)
Supplement: Supplementary file 3 — (DOC 27 kb) [file 10897_2015_9869_MOESM3_ESM.doc]

**Appendix** **A**  Recommendations for mammography and/or MRI for breast cancer unaffected women as stated in the Dutch Breast Cancer guideline^a^

*Lifetime breast cancer risk <20%*

- From 50 to and including 75 years of age, biennial mammography screening in the National Screening program.

*Lifetime breast cancer risk 20-30%*

- From 40 to 50 years of ages, annual mammography requested by GP.

- From 50 to and including 75 years of age, biennial mammography screening in the National Screening program.

*Lifetime breast cancer risk 30-40%*

- From 35 to 60 years of age, annual mammography and clinical breast examination.

- From 60 to and including 75 years of age, biennial mammography screening in the National Screening program.

*BRCA1/2 carriers and those at 50% risk of being a BRCA1/2 carrier*

*-* From 25 to 60 years of age, annual MRI and clinical breast examination.

- From 30 to 60 years of age, annual mammography.

- From 60 to 75 years of age, biennial mammography.

- After preventive prophylactic bilateral mastectomy no indication for surveillance.

^a^ Dutch Breast Cancer Guideline (CBO, 2008)
